# Moderate Mechanical Stimulation Protects Rats against Osteoarthritis through the Regulation of TRAIL via the NF-*κ*B/NLRP3 Pathway

**DOI:** 10.1155/2020/6196398

**Published:** 2020-05-23

**Authors:** Yue Yang, Yang Wang, Yawei Kong, Xiaoning Zhang, He Zhang, Xinyuan Feng, Ziyuan Wang, Peng Gao, Mingyue Yan, Lunhao Bai, Feng Li

**Affiliations:** ^1^Department of Orthopedic Surgery, Shengjing Hospital of China Medical University, Shenyang 110000, China; ^2^Department of Ultrasound, Shengjing Hospital of China Medical University, Shenyang 110000, China; ^3^International Patient Center, Brigham and Women's Hospital, Harvard Medical School, Boston 02115, USA; ^4^Department of Cell Biology, China Medical University, Shenyang 110000, China

## Abstract

The aim of this study was to examine exercise-related genes in articular cartilage identified through bioinformatics analysis to dissect the potential signaling pathway involved in mechanical stimulation in osteoarthritis (OA). To this end, we evaluated the GSE74898 dataset from the Gene Expression Omnibus database for exercise-related differentially expressed miRNAs (DE-miRNAs) using the R software package and predicted potential target genes for these miRNAs using miRTarBase. Functional annotation and pathway enrichment analysis were performed for these potential DE-miRNA targets. The effects of mechanical stimulation on the tumor necrosis factor-related apoptosis-induced ligand (TRAIL)/nuclear factor-kappa B (NF-*κ*B)/nucleotide-binding and oligomerization domain-like receptor containing protein 3 (NLRP3) signaling pathway were evaluated in articular cartilage and chondrocytes. A total of 394 DE-miRNAs were identified (103 upregulated miRNAs; 291 downregulated miRNAs) in the cartilage of rats following treadmill exercise compared to the cartilage of unexercised control rats. Thus, mechanical stimulation could modulate the TRAIL/NF-*κ*B/NLRP3 signaling pathway on OA. Histological and protein analysis demonstrated that moderate-intensity treadmill exercise could ameliorate OA through the downregulation of TRAIL. Furthermore, moderate cyclic tensile strain (CTS) could rescue chondrocytes from the effects of TRAIL via the inhibition of the nuclear translocation of NF-*κ*B p65 and formation of NLRP3. Our findings indicate that moderate mechanical stimulation could ameliorate the degeneration of cartilage and chondrocyte damage through the inhibition of the TRAIL/NF-*κ*B/NLRP3 pathway.

## 1. Introduction

Osteoarthritis (OA) is a whole joint disease that involves structural alterations in the articular cartilage, subchondral bone, ligaments, capsule, synovium, and periarticular muscles [1]. It affects a large portion of the population worldwide [2, 3]. Mechanical loading-based interventions are critical elements in OA treatment plans [1, 4–6].

Chondrocytes play a central role in maintaining cartilage homeostasis [7, 8]. They are mechanically sensitive and can adapt to the level of mechanical stimuli [9]. Chondrocyte mechanotransduction signaling pathways are complex, and the mechanisms by which chondrocytes convert mechanical stimuli into biochemical signals need further investigation.

MicroRNAs (miRNAs) play a role in the regulation of many biological processes [10–12]. Many miRNAs contribute to the chondrocyte phenotype via signaling pathways [13, 14]. Based on miRTarBase, gene ontology (GO) annotation, and Kyoto Encyclopedia of Genes and Genomes (KEGG) pathway enrichment analysis (GSE74898), we predicted that the signaling pathway involving tumor necrosis factor-related apoptosis-induced ligand (TRAIL), nuclear factor-kappa B (NF-*κ*B), and nucleotide-binding and oligomerization domain-like receptor containing protein 3 (NLRP3) played a key role in the mechanism by which mechanical stimulation ameliorates OA.

In the present study, we evaluated TRAIL expression levels in OA articular cartilage in rats following treadmill exercise compared to unexercised control animals. We also investigated changes in the NF-*κ*B/NLRP3 signaling pathway following the treatment of chondrocytes with mechanical stress in combination with TRAIL as a potential mechanism for the chondroprotective effect of moderate mechanical stimulation in OA.

## 2. Materials and Methods

### 2.1. miRNA Microarray

In the discovery step of this study, we used datasets that compared the miRNA expression of exercise-related genes in cartilage. The titles and abstracts of these datasets were screened, and those of interest were further investigated. Based on this analysis, the GSE74898 dataset was selected for further study. This dataset, which is based on the GPL6247 platform (Affymetrix Rat Gene 1.0 ST Array), contained three healthy Sprague-Dawley (SD) rats without exercise and 12 healthy Sprague-Dawley (SD) with exercise for a different number of days (2, 5, and 15 days) and was downloaded from the National Center for Biotechnology Information (NCBI) GEO database (https://www.ncbi.nlm.nih.gov/geo).

### 2.2. Prediction of Target Genes and miRNA-Gene Network Construction

The potential target genes of the identified DE-miRNAs were predicted using miRTarBase (http://mirtarbase.mbc.nctu.edu.tw/php/index.php), which is an experimentally validated micro-RNA-target interaction database [12]. The target genes were mapped in the STRING database (http://string-db.org) to assess the functional associations among these target genes [15]. Only interactions with a combined score of greater than 0.4 were considered as significant.

### 2.3. GO and Pathway Analysis

The database for annotation, visualization, and integrated discovery (DAVID 6.8, http://david-d.ncifcrf.gov/) was used to perform functional annotation and pathway enrichment analysis (GO and KEGG pathway analysis [16]) for the predicted targets of the selected DE-miRNAs. A *p* value of less than 0.05 was considered statistically significant.

### 2.4. Experimental Animals

Fifty male Sprague-Dawley (SD) rats (230 ± 10 g; specific-pathogen-free) were obtained from HFK Bioscience Co. Ltd. (Beijing, China). This study was approved by the Ethics Committee of Shengjing Hospital of China Medical University (no. 2017PS237K). The maintenance and care of the experimental rats followed the guidelines of the committee as previously described [17–19].

### 2.5. OA Model and Treadmill Running Protocols

**Figure 1:**
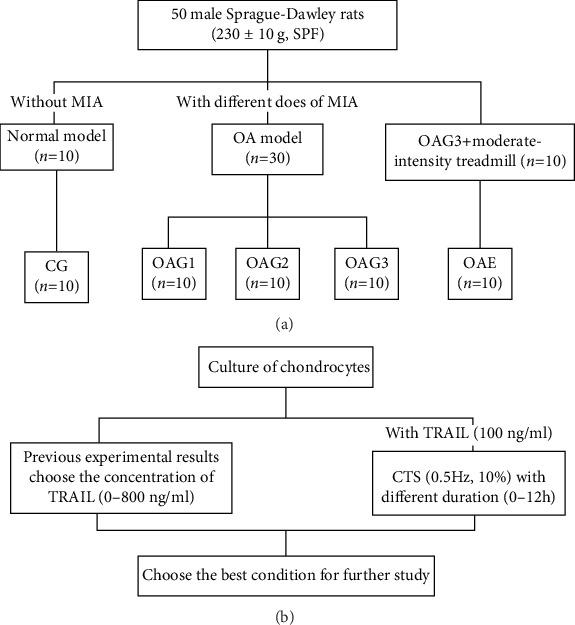
The design of the treatment schedule. (a) The design of the animal experiment. Experimental groups: CG: control group; OAG1, OAG2, and OAG3: OA groups injected with different doses of MIA; OAE: OAG3 subjected to moderate-intensity treadmill exercise. (b) The design of the chondrocyte experiment. CTS: cyclic tensile strain; TRAIL: tumor necrosis factor-related apoptosis-induced ligand; MIA: monoiodoacetate; OA: osteoarthritis; SPF: specific-pathogen-free.

The OA model and adaptive treadmill exercise protocols used in this study were based on previous studies [17–19]. After the adaptive treadmill exercise, the rats were randomly divided into five study groups (*n* = 10 in each group): control group (CG): intra-articular injection of 50 *μ*l sterile saline per cavity; OAG1, OAG2, and OAG3: intra-articular injection of 0.2 mg, 0.5 mg, or 1.0 mg MIA, respectively, per cavity in 50 *μ*l sterile saline; and OAE: OAG3 conditions with moderate treadmill exercise. The rats in groups CG, OAG1, OAG2, and OAG3 were kept sedentary, whereas the rats in group OAE began the treadmill exercise program 24 h after injection of MIA. These rats exercised at a speed of 12 m/min for 45 min once daily for five days per week for four weeks with appropriate photostimulation, acoustic stimulation, and electric stimulation (Figure 1).

### 2.6. Sampling and Tissue Preparation

After the last formal treadmill exercise session, the animals were anesthetized with 1.5% pentobarbital sodium 0.2 ml/100 g, intraperitoneal injection. The collection of serum, intra-articular lavage fluid (IALF), and articular cartilage was performed as previously described [17–19].

### 2.7. Histology

The method for hematoxylin and eosin and toluidine blue staining was performed as previously described [17–19]. Injury to the articular cartilage in the femur and tibia was assessed independently using the modified Mankin score [20] with a scale of 0 to 14 and the Osteoarthritis Research Society International (OARSI) score with a scale of 0 to 24 [21]. The tibial and femoral cartilage scores were then added, such that the maximum possible Mankin score was 28 and the maximum OARSI score was 48. Two experienced observers (Yue Yang and Xiaoning Zhang) performed the scoring in a blinded manner.

### 2.8. Immunohistochemistry

Immunohistochemistry was performed as previously described [17–19]. Collagen II levels are presented relative to CG. MMP-13 and NF-*κ*B p65 were expressed as the percentage of positive cells.

### 2.9. Enzyme-Linked Immunosorbent Assay (ELISA)

TNF-*α* and IL-1*β* levels in the knee IALF and serum were determined using ELISA kits (Tongwei, Shanghai, China) according to the manufacturer's instructions. The protein content in the IALF was measured to ensure that the ratio of dilution was equal.

### 2.10. Western Blotting

The primary antibodies are as follows: rabbit polyclonal anti-collagen II antibody (ab34712, 1 : 5,000; Abcam), molecular weight 142 kDa; rabbit monoclonal anti-NLRP3 antibody (ab210491, 1 : 1,000; Abcam), molecular weight 118 kDa; rabbit polyclonal anti-NF-*κ*B p65 antibody (AB21014, 1 : 500; AbSci), molecular weight 65 kDa; rabbit monoclonal anti-caspase-1 antibody (ab108362, 1 : 5,000; Abcam), molecular weight 45 kDa; rabbit monoclonal anti-pro caspase-1 antibody (ab179515, 1 : 1,000; Abcam), molecular weights 45 kDa and 42 kDa; mouse monoclonal anti-*β*-actin (60008-1-lg, 1 : 5,000, Proteintech Group), molecular weight 42 kDa; rabbit monoclonal anti-I*κ*B-*α* antibody (ab32518, 1 : 10,000; Abcam), molecular weight 36 kDa; rabbit polyclonal anti-TRAIL antibody (ab231063, 2 *μ*g/ml; Abcam), molecular weight 33 kDa; rabbit polyclonal anti-IL-1*β* antibody (ab150777, 1 : 1000; Abcam), molecular weight 31 kDa; and rabbit polyclonal anti-histone H2A.X (AB41012, 1 : 1,000, AbSci), molecular weight 19 kDa. *β*-Actin and histone H2A.X were used as internal controls. The processes of western blotting are consistent with previous articles [17–19].

### 2.11. Isolation and Culture of Chondrocytes

The isolation and culture of chondrocytes were performed as previously described [17].

### 2.12. Cell Counting Kit-8 (CCK-8) Assay

Primary rat chondrocytes were seeded in 96-well plates (5 × 10^3^ cells per well) and cultured for one day at 37°C in DMEM culture medium containing 10% FBS until 70 to 80% confluency. The medium was changed to serum-free DMEM containing increasing concentrations of TRAIL (abs01000, 0, 25, 50, 100, 200, 400, and 800 ng/ml), and cells were cultured for 12 h at 37°C. After TRAIL treatment, 10 *μ*l CCK-8 (Beyotime, C0042) and 90 *μ*l serum-free DMEM were added to each well followed by incubation for 2 h at 37°C. The absorbance at 450 nm was measured using a spectrophotometer (Synergy H1, BioTek, USA).

### 2.13. Exposure of Chondrocytes to CTS

Chondrocytes were grown on collagen I-coated BioFlex 6-well culture plates (Flexcell International, Hillsborough, NC) to 80% to 90% confluency. CTS experiments were performed using the FX-5000 Flexcell system (Flexcell International, McKeesport, PA). The plates were placed on a loading station in an incubator with 5% CO_2_ such that when the vacuum was applied to the loading station, the membrane deformed across the post face, creating uniform biaxial strain. Chondrocytes were subjected to CTS (10%, 0.5 Hz) for 0, 1, 2, 4, 8, or 12 h in the presence of 100 ng/ml TRAIL. The stimulation of CTS and TRAIL on chondrocytes began at the same time. A 4 h treatment with CTS was determined to be the optimal duration and was used for subsequent experiments. All chondrocytes were harvested at 12 h with TRAIL.

### 2.14. Quantitative Real-Time Polymerase Chain Reaction (qPCR)

The processes of qPCR are consistent with previous articles [19]. Expression levels were calculated by the 2^-*ΔΔ*CT^ method [22] using *β*-actin as the reference gene. The primer pair sequences were specific to rat MMP-13 (F-5′-TGATGATGAAACCTGGACAAGCA-3′; R-5′-GAACGTCATCTCTGGGAGCA-3′), MMP-1 (F-5′-TGTTCGCCTTCTACAGAGGAGACC-3′; R-5′-TGTCGGTCCACGTCTCATCCAG-3′), and *β*-actin (F-5′-GGAGATTACTGCCCTGGCTCCTA-3′; R-5′-GACTCATCGTACTC CTGCTTGCTG-3′).

### 2.15. Cellular ROS Production

**Figure 2:**
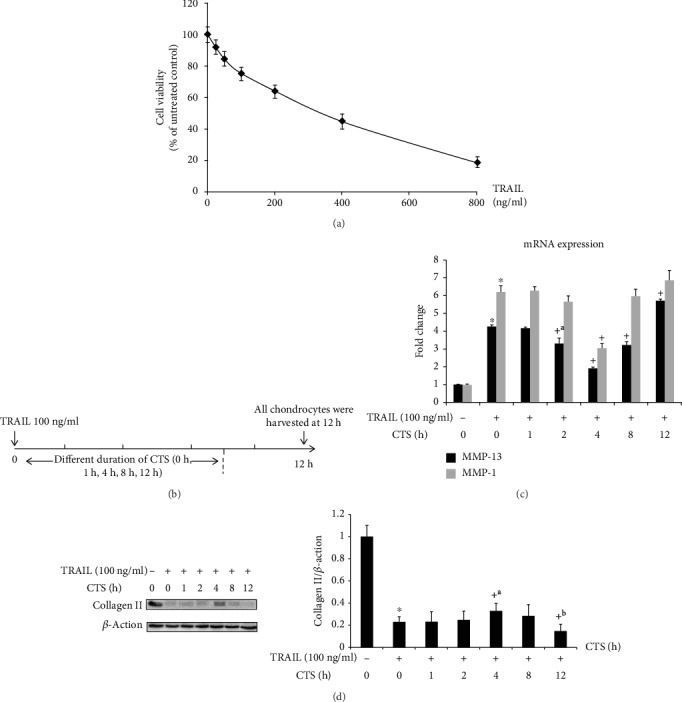
Western blot and qPCR analysis of chondrocytes treated with CTS for different durations. (a) The results of CCK-8 test. (b) The design of chondrocytes was subjected to CTS (10%, 0.5 Hz) for different durations (0 h, 1 h, 2 h, 4 h, 8 h, and 12 h) with TRAIL. (c) MMP-13 and MMP-1 mRNA expression in chondrocytes following different durations of CTS (10%, 0.5 Hz) was determined by qPCR. Differences between untreated and TRAIL (100 ng/ml)-induced chondrocytes (^∗^*p* < 0.001) and TRAIL-induced chondrocytes and those subjected to CTS for different durations (^+^*p* < 0.001, ^+a^*p* = 0.001) were significant (ANOVA). Data are presented as the mean ± 95%confidence intervals; *n* = 9 per group. (d) Collagen II protein levels in chondrocytes. Differences between untreated and TRAIL (100 ng/ml)-induced chondrocytes (^∗^*p* < 0.001) and TRAIL-induced chondrocytes and those subjected to CTS of different durations (^+^*p* < 0.001, ^+a^*p* = 0.006, and ^+b^*p* = 0.002) were significant (ANOVA). *β*-Actin was used as an internal control. Data are presented as the mean ± 95%confidence intervals; *n* = 3 per group.

Chondrocytes were seeded in 6-well plates (1.5 × 10^6^ cells/well). Chondrocytes were exposed to different doses of TRAIL (abs01000, 25, 50, or 100 ng/ml) for 12 h with or without CTS (10%, 0.5 Hz, 4 h). All chondrocytes were harvested at 12 h with TRAIL (Figure 2(b)). ROS production was measured using 2′,7′-dichlorodihydrofluorescein diacetate (DCFH-DA) (S0033, Beyotime),s which is directly oxidized by ROS (e.g., superoxide ion, hydrogen peroxide, and hydroxyl). Chondrocytes were incubated with 10 *μ*M DCFH-DA for 45 min at 37°C in the dark and then washed three times in PBS. Fluorescence was detected by fluorescent microscopy and measured with the BD FACSCalibur (488 nm, excitation; 525 nm, emission).

### 2.16. Detection of Apoptosis by Flow Cytometry

Chondrocytes were washed three times with PBS, resuspended in 500 *μ*l binding buffer, and collected as described above. The cells were double-stained using the Annexin V-FITC/propidium iodide (PI) staining kit (AD101; Dojindo, Japan), which is a standard method to distinguish between cells dying via apoptosis or necrosis. The total percentage of the apoptotic cells is the sum of both the early and late stages of apoptosis (Annexin V-FITC positive). The cells that stained only with PI were considered the necrotic cell population. Data acquisition and analysis were performed using CellQuest software (BD Biosciences, San Jose, CA, USA).

### 2.17. Immunofluorescence Analysis of Chondrocytes

Immunofluorescence was performed as previously described [17].

### 2.18. Statistical Analysis

Data are expressed as the means with 95% confidence intervals (CIs) and analyzed using SPSS statistical software version 22 (SPSS Inc., Chicago, IL, USA). Shapiro-Wilk and Levene's tests were applied to evaluate the normality and homogeneity of the results, respectively. For variables that exhibited normal distribution, data were analyzed using one-way analysis of variance (ANOVA). A *p* value of less than 0.05 indicated statistical significance.

## 3. Results

### 3.1. Identification of DE-miRNAs and Their Target Genes

Analysis of the GSE74898 miRNA array identified a total of 565, 430, and 618 miRNAs that were differentially expressed more than twofold in the cartilage of unexercised rats compared to that of rats exercised for 2, 5, or 15 days, respectively. A total of 394 DE-miRNAs were common across the three time points, including 103 upregulated and 291 downregulated miRNAs. The volcano plot and Venn diagram for these DE-miRNAs are presented in Fig. S1.

### 3.2. GO Functional Enrichment Analysis

Four categories of GO functional annotation analysis were performed on the potential target genes, including molecular functions, biological processes, cellular components, and biological pathways. As shown in Fig. S2, the most enriched GO functions for these DE-miRNA target genes included motor activity, signal transduction, extracellular, and the TRAIL signaling pathway in these four categories, respectively.

### 3.3. KEGG Pathway Enrichment Analysis

KEGG pathway enrichment analysis was conducted to further analyze the enriched pathways for these target genes. The enriched KEGG pathways included the B cell receptor signaling pathway, cell cycle, Fc gamma R-mediated phagocytosis, systemic lupus erythematosus, Fc epsilon RI signaling pathway, oocyte meiosis, NF-kappa B signaling pathway, viral carcinogenesis, p53 signaling pathway, and chemokine signaling pathway (Fig. S3B). We predicted the involvement of the TRAIL signaling pathway in the exercise-induced changes in cartilage through the GO functional enrichment analysis. Because TRAIL can activate the NF-*κ*B signaling pathway, it could be the link between mechanical stimulation and the responses of chondrocytes to proinflammatory cytokines [8]. Thus, the TRAIL/NF-*κ*B signaling pathway may play a role in the effects of mechanical stimulation on OA.

### 3.4. Histological and Immunohistochemical Analysis

The results of histological assessment (Mankin and OARSI scores) and collagen II are consistent with previous results with different concentrations [17] (data not shown).

**Figure 3:**
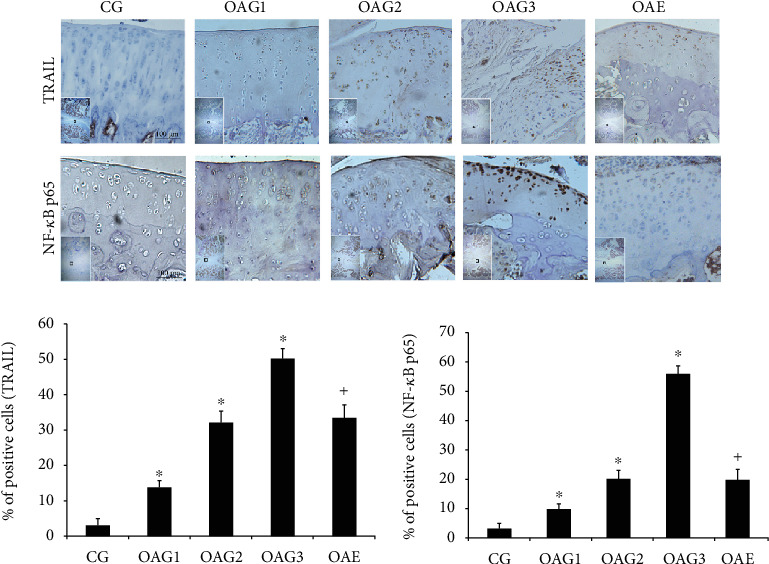
Immunohistochemical staining. The micrographs show the percentages of TRAIL-positive cells and the percentage of NF-*κ*B p65 nuclear translocation in the articular cartilage of each experimental group. Differences between CG and OAG1, OAG2, and OAG3 (^∗^*p* < 0.001) and OAG3 and OAE (^+^*p* < 0.001) were significant (ANOVA). Data are presented as the mean ± 95%confidence intervals; *n* = 5 rats per group. Experimental groups: CG: control group; OAG1, OAG2, and OAG3: OA groups treated with different doses of MIA; OAE: OAG3 subjected to moderate-intensity treadmill exercise.

Immunohistochemical staining (TRAIL and NF-*κ*B p65) has similar results with Mankin and OARSI scores (the percentages of TRAIL-positive cells: CG = 3.2, 95% CI 1.4-4.9; OAG1 = 13.9, 95% CI 12.2-15.6; OAG2 = 32.3, 95% CI 29.3-35.3; OAG3 = 50.3, 95% CI 47.4-53.1; and OAE = 33.4, 95% CI 29.9-36.9; the percentage of NF-*κ*B p65 nuclear translocation: CG = 3.1, 95% CI 1.4-4.9; OAG1 = 9.9, 95% CI 8.3-11.5; OAG2 = 20.0, 95% CI 18.0-22.0; OAG3 = 55.9, 95% CI 50.7-61.1; and OAE = 19.7, 95% CI 16.4-23.0) (Figure 3).

### 3.5. TNF-*α* and IL-1*β* ELISA

The results of TNF-*α* and IL-1*β* are consistent with previous results with different concentrations [17] (data not shown).

### 3.6. Western Blot Analysis

The results of collagen II and NF-*κ*B p65 in the cartilage are consistent with previous results with different concentrations [17] (data not shown).

**Figure 4:**
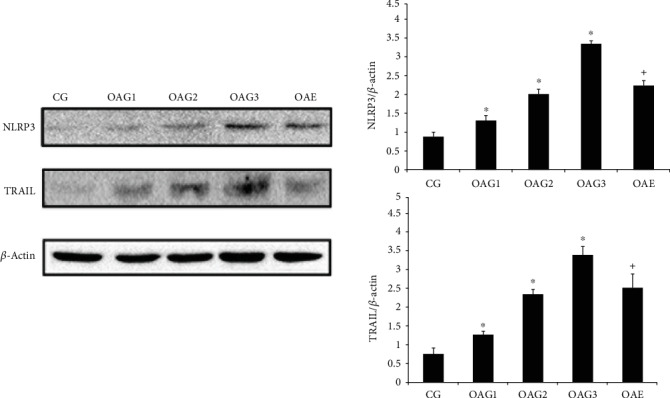
Protein expression levels were determined by western blotting of total protein extracted from cartilage. Differences between CG and OAG1, OAG2, and OAG3 (^∗^*p* < 0.001) and OAG3 and OAE (^+^*p* < 0.001) were significant (ANOVA). *β*-Actin was used as internal controls. Data are presented as the mean ± 95%confidence intervals; *n* = 3 rats per group. Experimental groups: CG: control group; OAG1, OAG2, and OAG3: OA groups treated with different doses of MIA; OAE: OAG3 subjected to moderate-intensity treadmill exercise.

The changes in the levels of NLRP3 and TRAIL in the cartilage between the different groups paralleled those observed by histology and immunohistochemistry (Figure 4).

**Figure 5:**
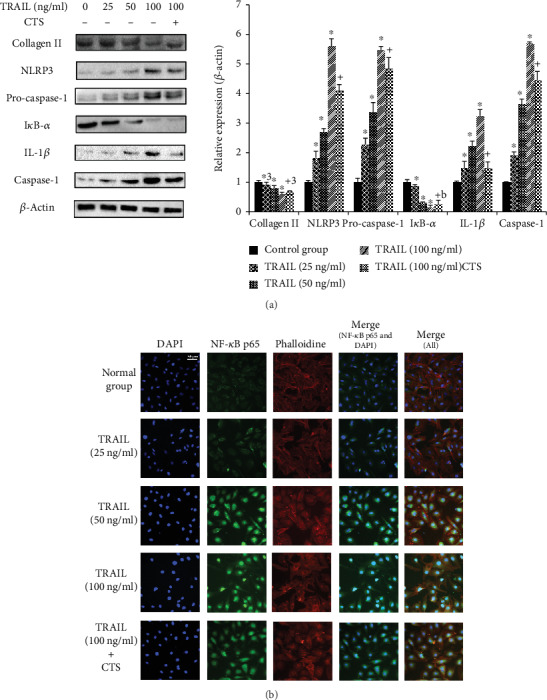
Western blot and immunofluorescence analysis of chondrocytes. (a) Western blotting results for collagen II, NLRP3, procaspase-1, I*κ*B-*α*, IL-1*β*, and caspase-1. Differences between untreated and TRAIL (0, 25, 50, and 100 ng/ml)-induced chondrocytes (^∗^*p* < 0.001, ^∗a^*p* = 0.005) and TRAIL (100 ng/ml)-induced chondrocytes and those subjected to CTS for 4 h (^+^*p* < 0.001, ^+a^*p* = 0.003, and ^+b^*p* = 0.001) were significant (ANOVA). Data are presented as the mean ± 95%confidence intervals; *n* = 3 per group. (b) Effects of CTS for 4 h on the nuclear translocation of NF-*κ*B p65 in TRAIL-induced chondrocytes. The chondrocytes were immunostained using anti-NF-*κ*B p65 rabbit antibody (green) and visualized by confocal microcopy. The cytoskeleton was visualized with phalloidin (red), and the cell nucleus was stained with 4,6-diamidino-2-phenylindole (DAPI; blue). Scale bar, 50 *μ*m.

TRAIL induced a significant decrease in collagen II compared to untreated chondrocytes, but the collagen II levels increased in the chondrocytes following a 4 h treatment with CTS (Figure 2(d)). TRAIL caused a dose-dependent decrease in collagen II and I*κ*B-*α* protein levels and a dose-dependent increase in NLRP3, procaspase-1, IL-1*β*, and caspase-1 protein levels in chondrocytes. However, CTS treatment for 4 h ameliorated these changes (Figure 5(a)).

### 3.7. qPCR Assay

The relative expression levels of MMP-13 and MMP-1 in chondrocytes are shown in Figure 2(c). TRAIL caused an increase in the expression of both MMP-13 and MMP-1; however, these increased levels were decreased by 4 h treatment with CTS (relative expression levels of MMP-1: control = 1.00, 95% CI 0.97-1.03; TRAIL only = 6.19, 95% CI 5.83-6.54; TRAIL+CTS 1 h = 6.25, 95% CI 5.99-6.51; TRAIL+CTS 2 h = 5.65, 95% CI 5.32-5.97; TRAIL+CTS 4 h = 3.05, 95% CI 2.82-3.28; TRAIL+CTS 8 h = 5.95, 95% CI 5.53-6.37; and TRAIL+CTS 12 h = 6.81, 95% CI 6.22-7.41; relative expression levels of MMP-13: control = 1.00, 95% CI 0.99-1.01; TRAIL only = 4.34, 95% CI 4.12-4.36; TRAIL+CTS 1 h = 4.16, 95% CI 4.08-4.24; TRAIL+CTS 2 h = 3.30, 95% CI 2.99-3.61; TRAIL+CTS 4 h = 1.89, 95% CI 1.79-1.98; TRAIL+CTS 8 h = 3.25, 95% CI 3.12-3.38; and TRAIL+CTS 12 h = 5.71, 95% CI 5.60-5.81). These data were consistent with the collagen II results.

### 3.8. Immunofluorescence in Chondrocytes

As shown in Figure 5(b), TRAIL caused significant accumulation of NF-*κ*B p65 protein in the nuclei of chondrocytes compared to the control group. CTS suppressed the nuclear translocation of NF-*κ*B p65 induced by TRAIL (Figure 5(b)).

### 3.9. ROS Analysis of Chondrocytes

**Figure 6:**
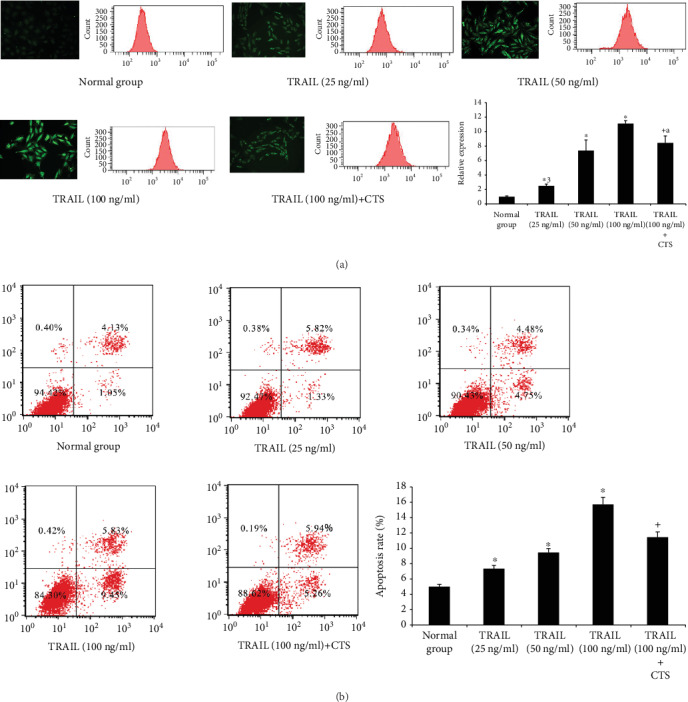
Reactive oxygen species (ROS) and apoptosis in chondrocytes. (a) Fluorescence microscopy and flow cytometry of ROS in chondrocytes. Differences between untreated and TRAIL (0, 25, 50, and 100 ng/ml)-induced chondrocytes (^∗^*p* < 0.001, ^∗a^*p* = 0.012) and TRAIL (100 ng/ml)-induced chondrocytes and those subjected to CTS for 4 h (^+a^*p* = 0.012) were significant (ANOVA). Data are presented as the mean ± 95%confidence intervals; *n* = 3 per group. (b) Chondrocyte apoptosis analysis by flow cytometry. Differences between untreated and TRAIL (0, 25, 50, and 100 ng/ml)-induced chondrocytes (^∗^*p* < 0.001) and TRAIL (100 ng/ml)-induced chondrocytes and those subjected to CTS for 4 h (^+^*p* < 0.001) were significant (ANOVA). Data are presented as the mean ± 95%confidence intervals; *n* = 3 in each group.

ROS production in chondrocytes was enhanced by TRAIL in a dose-dependent manner (Figure 6(a)). CTS significantly reduced the ROS levels induced by TRAIL (relative intensity of ROS: normal group = 1.00, 95% CI 0.94-1.07; TRAIL (25 ng/ml) = 2.55, 95% CI 2.36-2.74; TRAIL (50 ng/ml) = 7.82, 95% CI 5.41-10.23; TRAIL (100 ng/ml) = 10.64, 95% CI 8.33-12.95; and TRAIL (100 ng/ml)+CTS = 8.50, 95% CI 7.56-9.44).

### 3.10. Analysis of Chondrocyte Apoptosis

The percentage of apoptotic chondrocytes significantly increased in response to treatment will increase the amounts of TRAIL. These effects were abrogated by moderate CTS (Figure 6(b)) (the percentage of apoptotic chondrocytes: normal group = 5.04, 95% CI 4.68-5.40; TRAIL (25 ng/ml) = 7.37, 95% CI 6.84-7.91; TRAIL (50 ng/ml) = 9.46, 95% CI 8.92-9.99; TRAIL (100 ng/ml) = 15.70, 95% CI 14.74-16.66; and TRAIL (100 ng/ml) + CTS = 11.49, 95% CI 10.86-12.12).

## 4. Discussion

Exercise is one of the most widely accepted nonpharmacological therapies for OA [23]. However, how exercise affects the pathology of OA is still unclear. As mechanosensitive cells, chondrocytes synthesize the extracellular matrix and depend on intracellular signals generated in response to biomechanical stress [24, 25]. Therefore, it is important to understand the mechanisms underlying the mechanical stimulation of cartilage and chondrocytes. miRNAs are a group of small, endogenous noncoding RNAs that possess a variety of biological functions. Recent studies showed that miRNAs are involved in the regulation of gene expression in cartilage during exercise [15, 16]. In this study, we identified exercise-related miRNAs in rat cartilage through the analysis of GEO datasets. GO and KEGG pathway analysis revealed that the TRAIL/NF-*κ*B/NLRP3 signaling pathway may be of importance.

There were several principal findings in this study. First, there were significant differences observed in the articular cartilage of the knees of rats injected with MIA to induce OA compared to control rats. Exercise had significant therapeutic effects against the MIA-induced OA. Second, the TRAIL levels in the chondrocytes were substantially increased by MIA in a dose-dependent manner. Moderate CTS reduced the inflammatory response induced by TRAIL in the chondrocytes. Third, the suppression of the TRAIL-induced inflammatory response by moderate CTS appeared to occur through the NF-*κ*B/NLRP3 pathway.

To study the connection between mechanical stress and OA progression, we investigated the effect of exercise on an OA rat model induced by the intra-articular injection of MIA. Our results indicate that there were significant differences in both the histology (Mankin and OARSI scores) and collagen II, NLRP3, TRAIL, and NF-*κ*B p65 protein levels between the articular cartilage of the knees of MIA-treated and control rats. Synovitis plays a key role in the pathogenesis of OA and is thought to contribute to cartilage degeneration [7]. Synovitis generates inflammatory mediators (TNF-*α* and IL-1*β*) that are released into the synovial cavity. We found that moderate exercise of rats injected with MIA not only ameliorated the histological changes but also relieved the damage of collagen II in the cartilage and decreased the levels of these inflammatory mediators in the serum and IALF from these rats. Together, our results corroborate the finding that exercise alleviates damage in the OA knee, which occurs through the suppression of TRAIL, NF-*κ*B p65, and NLRP3 levels in the cartilage.

The mechanisms by which chondrocytes convert biomechanical signals into intracellular events have become an area of intense interest in OA research. Thus, we investigated a cellular model using CTS on chondrocytes. Our previous study showed that CTS (10%, 0.5 Hz) for 4 h alleviated the chondrocyte damage induced by IL-1*β* [17]. In addition, our findings in the current indicate that CTS administered for different lengths of time could have different effects on TRAIL-induced chondrocytes with 4 h CTS increasing collagen II levels and alleviating chondrocyte damage.

The present findings are consistent with the observations that treadmill exercise causes histological changes in articular cartilage. These findings further suggest that mechanical stimulation is a critical determinant for chondrocytes. Accumulating evidence suggests that TRAIL is a pivotal mediator of OA. The TRAIL signaling pathway can induce apoptosis, leading to joint inflammation and cartilage destruction. Furthermore, we found that the levels of TRAIL increased in OA cartilage. Interestingly, the TRAIL signaling pathway can induce apoptosis in normal cells [26]. Accumulating evidence has demonstrated that articular chondrocytes can be eliminated by apoptosis in OA. The NLRP3 inflammasome produces proinflammatory cytokines, which cause cartilage degeneration and synovial inflammation, and has been implicated in apoptosis in OA [27]. Nuclear translocation of NF-*κ*B p65 is a key event in the activation of this inflammasome [28, 29]. Thus, we investigated the NF-*κ*B/NLRP3 signal on chondrocytes induced by TRAIL.

Intriguingly, the effects of TRAIL on chondrocytes occur through the NF-*κ*B/NLRP3 signaling pathway. TRAIL can inhibit the expression of I*κ*B-*α* and accelerate NF-*κ*B p65 nuclear translocation. There is also evidence suggesting that the NLRP3 inflammasome is involved in TRAIL-induced chondrocytes, leading to cartilage degradation and synovial inflammation through the activation of NF-*κ*B signaling. Our results showed that TRAIL could also increase ROS generation, which has been shown to activate NLRP3. The NLRP3 inflammasome is composed of NLRP3, adaptor apoptosis-associated speck-like protein containing a CARD, and procaspase-1. NLRP3 ultimately activates caspase-1, which causes maturation of proinflammatory cytokines (e.g., IL-1*β*) into their active forms and their secretion to further the inflammatory response [30]. IL-1*β* also stimulates chondrocytes to release MMP-13, which degrades collagen II in the extracellular matrix, leading to a loss of cartilage. Interestingly, NLRP3 is an indicator of pyroptosis, which mainly occurs in the early stage (or acute inflammatory response) of OA [31, 32]. NLRP3 protein levels were significantly higher in the early stage of OA, but NLRP3 would decrease at the end of OA [33].

Importantly, in this study, we found that the NF-*κ*B/NLRP3 signal transduction pathway is central to the effect of CTS. CTS reduced the level of ROS and inhibited NF-*κ*B p65 nuclear translocation via the activation of I*κ*B-*α*. The decrease in ROS could also inhibit the activation of the NLRP3 inflammasome. Indeed, moderate CTS could inhibit the activation of the NF-*κ*B/NLRP3 signaling pathway and block chondrocyte destruction caused by TRAIL, presumably by abrogating IL-1*β* production and collagen II breakdown in chondrocytes. Hence, the anti-inflammatory actions of CTS are mediated by the inhibition of both NF-*κ*B nuclear translocation and NLRP3 activation in TRAIL-induced chondrocytes. Moreover, the results of this study also showed that moderate biomechanical stimulation not only decreased the TRAIL levels in the cartilage but also reduced the sensitization of articular cartilage and chondrocytes to the inflammatory response. These results are consistent with previous studies [24].

This study had some limitations. First, we did not investigate the effects of a variety of CTS conditions on OA. Further study is needed to explore the effects of different CTS conditions (e.g., intensity and frequency) on articular cartilage and chondrocytes. Second, CTS is two-dimensional loading, and chondrocytes are strained in a monolayer in which only one surface is elongated. We will investigate different dimensions of CTS in future studies.

## 5. Conclusion

In summary, we used treadmill exercise of MIA-induced OA rats and CTS of chondrocytes to explore the effects of mechanical stimulation in OA. Our findings indicated that TRAIL increased during the progression of OA and could damage chondrocytes via the NF-*κ*B/NLRP3 pathway. Moderate mechanical stimulation may reduce the apoptosis of chondrocytes through the inhibition of this pathway. Our results provide not only crucial leads to unveiling the effects of mechanical stress on articular cartilage and chondrocytes but also molecular evidence for biochemical signals generated by mechanical stimulation.

## Data Availability

The data used to support the findings of this study have been deposited in the PMC6587477 (doi:10.1002/jcp.27592) and the supplementary information files.

## Supplementary Materials

Supplementary materials Figure S1: volcano plots and Venn diagram of the DE-miRNAs. (A-C) The black dots represent miRNAs that are not differentially expressed between the cartilage from unexercised or exercised rats for 2 (A), 5 (B), or 15 (C) days. The red and green dots represent the upregulated and downregulated miRNAs, respectively. (D) The Venn diagram of these DE-miRNAs. Figure S2: GO functional annotation analysis. Four categories of GO functional annotation analysis were performed on the potential target genes, including molecular function (A), biological process (B), cellular component (C), and biological pathway (D). Figure S3: protein-protein interaction (PPI) network and KEGG pathway enrichment analysis of target genes.
