# Supplementary material for: Moderate Mechanical Stimulation Protects Rats against Osteoarthritis through the Regulation of TRAIL via the NF-κB/NLRP3 Pathway
Source: Oxid Med Cell Longev. 2020 May 23;2020:6196398. doi: 10.1155/2020/6196398 (PMC7267856; doi:10.1155/2020/6196398)
Supplement: Supplementary materials — Figure S1: volcano plots and Venn diagram of the DE-miRNAs. (A-C) The black dots represent miRNAs that are not differentially expressed between the cartilage from unexercised or exercised rats for 2 (A), 5 (B), or 15 (C) days. The red and green dots represent the upregulated and downregulated miRNAs, respectively. (D) The Venn diagram of these DE-miRNAs. Figure S2: GO functional annotation analysis. Four categories of GO functional annotation analysis were performed on the potential target genes, including molecular function (A), biological process (B), cellular component (C), and biological pathway (D). Figure S3: protein-protein interaction (PPI) network and KEGG pathway enrichment analysis of target genes. [file 6196398.f1.pdf]

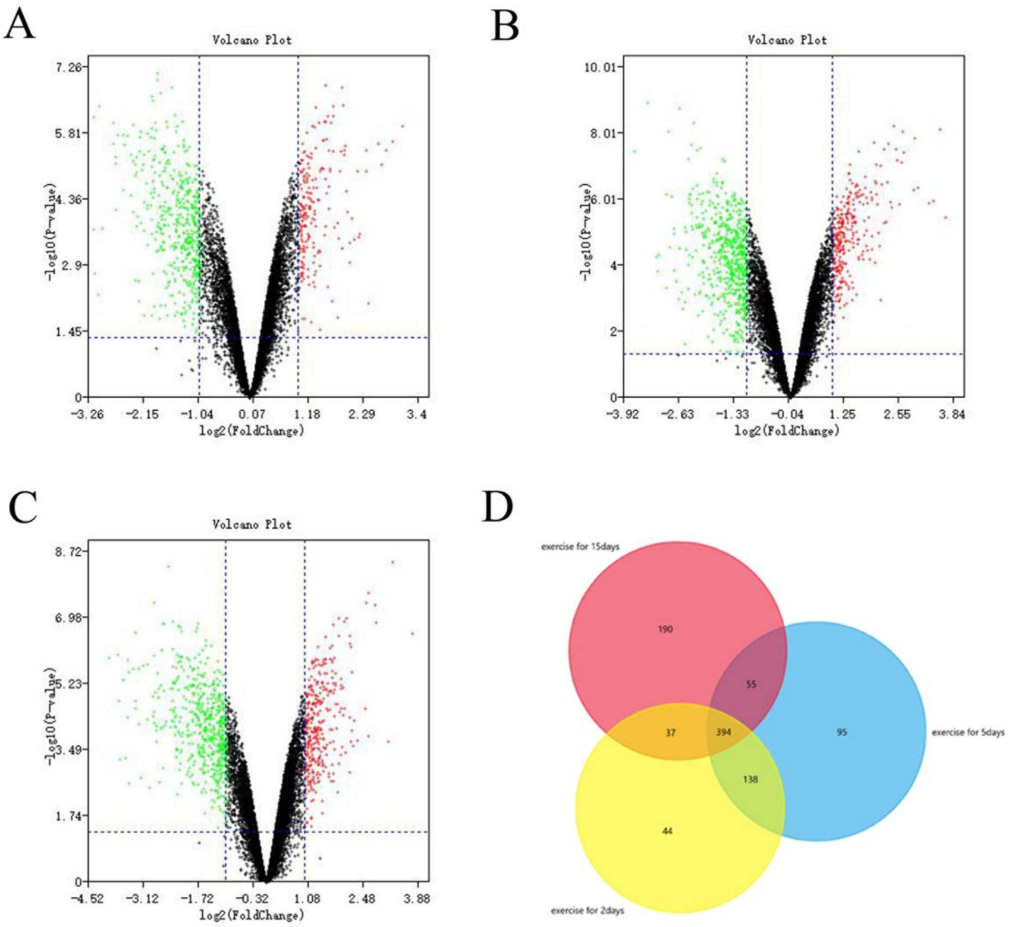

Fig S1. Volcano plots and Venn diagram of the DE-miRNAs. (A-C) The black dots represent miRNAs that are not differentially expressed between cartilage from unexercised or exercised rats for 2 (A), 5 (B), or 15 (C) days. The red and green dots represent the upregulated and downregulated miRNAs, respectively. (D) The Venn diagram of these DE-miRNAs..

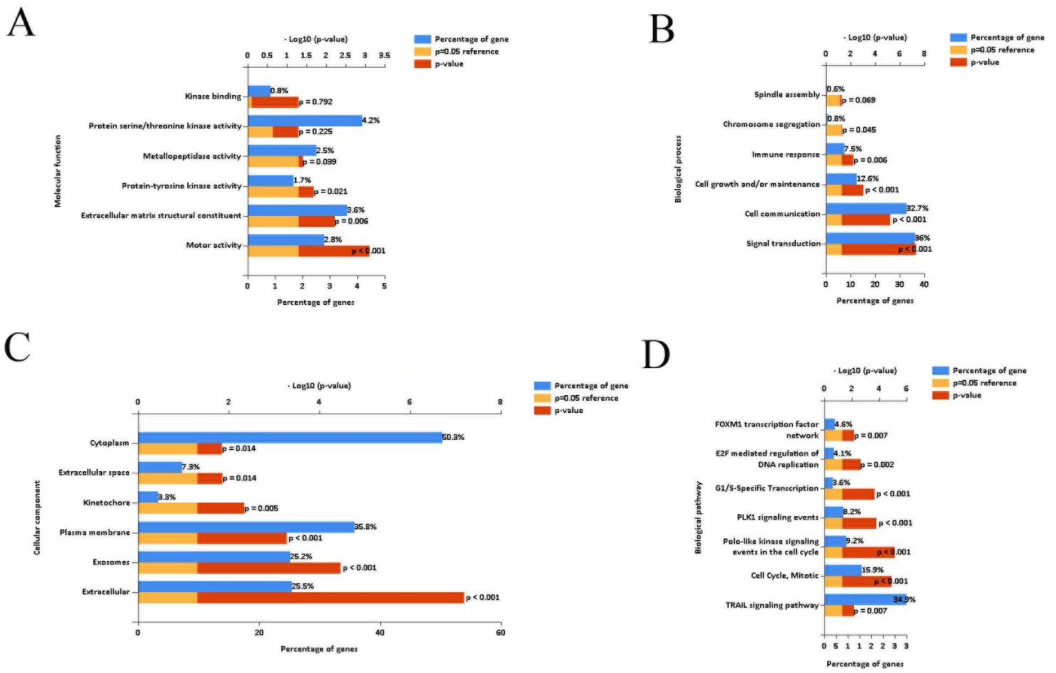

Fig S2. GO functional annotation analysis. Four categories of GO functional annotation analysis were performed on the potential target genes, including molecular function (A), biological process (B), cellular component (C), and biological pathway (D).

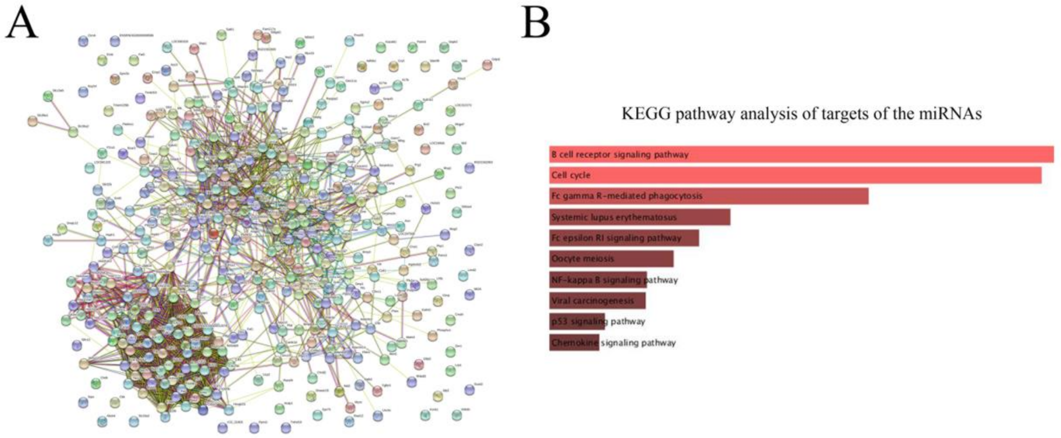

Fig S3. Protein-protein interaction (PPI) network and KEGG pathway enrichment analysis of target genes.
